# Laboratory Infrared Spectra and Band Strengths of Carbonyl Sulfide (OCS) in CH_3_OH- and CO-Rich Ice Mixtures for Analyzing Interstellar Ice Observations

**DOI:** 10.1021/acsearthspacechem.5c00134

**Published:** 2025-07-22

**Authors:** Katerina Slavicinska, Charlotte Coone, Bryce Benz, Harold Linnartz, A. C. Adwin Boogert, Ko-Ju Chuang

**Affiliations:** † Laboratory for Astrophysics, Leiden Observatory, 4496 Leiden University, P.O. Box 9513, Leiden 2300 RA, The Netherlands; ‡ Leiden Observatory, Leiden University, P.O. Box 9513, Leiden 2300 RA, The Netherlands; § Institute for Astronomy, University of Hawaii at Manoa, 2680 Woodlawn Drive, Honolulu, Hawaii 96822, United States

**Keywords:** infrared spectroscopy, interstellar ices, interstellar sulfur, astrochemistry, star-forming regions, protostars

## Abstract

Carbonyl sulfide (OCS) is currently the only securely detected sulfur-bearing species in interstellar ices, making it an ideal window into solid-state sulfur chemistry in dense star-forming regions. Previous astronomical observations of the OCS asymmetric stretching mode (ν_3_) at ∼2040 cm^–1^ (∼4.9 μm) demonstrate that interstellar OCS may be embedded in CH_3_OH-rich ices, indicating that OCS likely forms in the coldest, densest parts of star-forming regions where catastrophic CO freezeout occurs. However, a significant portion of the OCS ice observations cannot be fit with binary OCS:CH_3_OH laboratory ice mixtures alone, suggesting a greater degree of chemical complexity in the local ice environment. With this work, we aim to aid future studies of the abundance, physicochemical environment, and evolutionary history of interstellar OCS ice, now enabled for many more interstellar environments by the *James Webb* Space Telescope. We provide a library of new laboratory IR transmission spectra of the tetrahedron of the OCS in CH_3_OH- and CO-rich ice mixtures, some of which also include H_2_S and H_2_O. Of these new spectra, the tertiary OCS:CO:CH_3_OH ice mixtures provide the best fits to observations of high-mass protostars, providing further support for the hypothesis that the atom of the OCS forms with CH_3_OH, possibly via chemical pathways involving frozen-out CO. We calculate apparent band strengths of the ν_3_ mode in the OCS:CH_3_OH and the OCS:CO:CH_3_OH ice mixtures. The derived values are consistent (within uncertainties) with the apparent band strength of the feature in pure OCS ice, 1.2 × 10^–16^ cm molec^–1^. We therefore recommend using this value when quantifying interstellar OCS ice column densities.

## Graphical abstract

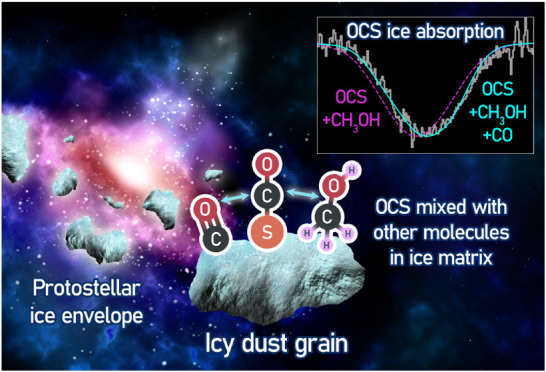

## Introduction

In the past two decades, sulfur-bearing ices have been a major focus of astrochemical research due to the observed gas-phase sulfur depletion in dense star-forming regions.^1–5^ However, of the many sulfur-bearing molecules proposed by such works to exist in interstellar ices, carbonyl sulfide (OCS) remains the only secure detection despite its low abundance (∼0.05–0.5% with respect to H_2_O).^6,7^ This is due to the high band strength (on the order of 10^–16^ cm molec^–1^)^8^ and the relatively isolated peak position (4.9 μm) of its strongest IR vibrational feature, the asymmetric stretching mode (ν_3_). These spectral properties aided its early first detection^9^ and subsequent identification as OCS^10^ in ground-based IR spectra of the ice envelope surrounding the massive protostar W33A. Since then, OCS has been detected toward the ice envelopes of over 20 massive protostars,^6,11–13^ as well as the ice envelope of one low-mass protostar^14^ and two background stars behind a dense molecular cloud.^7^

Although OCS is decidedly not a major sulfur carrier in dense star-forming regions, its strong ν_3_ absorption feature offers a unique window into the solid-state sulfur chemistry of such environments. Experiments presented in Palumbo et al.^10^ demonstrated that the profile of this band is highly dependent on the chemical composition of the surrounding ice matrix, and out of all of the laboratory ice mixtures compared to the observations, the ices rich in CH_3_OH provided the best fit to many of the observed absorptions.^6,11^ Additionally, in a survey of 23 massive protostars, Boogert et al.^6^ found that OCS ice column densities correlate well with the ice column densities of CH_3_OH, OCN^–^, and the polar CO component, while they correlate poorly with the ice column densities of H_2_O and the apolar CO component. A correlation between OCS and CH_3_OH column densities (i.e., small scatter in the OCS/CH_3_OH ratio) is also observed toward massive protostars in the gas phase.^15^ These results indicate that most OCS ice likely formed alongside CH_3_OH after the heavy CO freezeout stage in the densest regions of the clouds and envelopes, possibly via routes such as the sulfurization of CO^16^ or the addition of SH radicals to CO.^17^

Despite these indications that interstellar OCS ice may be embedded in a CH_3_OH-rich environment, the band strength of the ν_3_ feature in CH_3_OH-rich ices has not been measured. So far, most currently reported interstellar OCS ice column densities^6,11^ were calculated using an OCS ν_3_ band strength of 1.5 × 10^–16^ cm molec^–1^, derived for pure OCS ice using a refractive index measured for liquid OCS and a simply assumed ice density of 1 g cm^–3^.^18^ Recently, Yarnall and Hudson^8^ published an improved OCS ν_3_ band strength of 1.2 × 10^–16^ cm molec^–1^ for both pure OCS ice and OCS in a 1:20 binary mixture with H_2_O ice using new refractive index and density values that were measured directly for amorphous OCS ice. Nevertheless, existing OCS observations indicate that neither pure nor H_2_O-dominated OCS ices are relevant to the interstellar medium.

Furthermore, the large sample of OCS ice observations in Boogert et al.^6^ revealed that in a significant portion of the investigated sources, the observed OCS ν_3_ peak position is slightly red-shifted (by up to ∼0.01 μm) relative to the peak positions of OCS in binary laboratory ice mixtures with CH_3_OH. In contrast, comparably red-shifted peak positions of the OCS feature are observed in laboratory spectra of CO- and H_2_S-containing ices that were irradiated and then heated.^19,20^ Thus, the observed red shifts may indicate a higher degree of chemical complexity in the ice matrix in which OCS is embedded and could be interpreted as tentative, indirect evidence for OCS formation via energetic processing of other sulfur-bearing ices. However, a lack of publicly available laboratory IR spectra of OCS in more chemically complex ice matrices with carefully controlled ice mixing ratios precludes drawing more specific conclusions about the chemical environment of OCS toward these sources with red-shifted OCS features.

The present work aims to fill several gaps in experimental studies of the OCS ice studies. First, we present the transmission IR spectrum from 4000 to 500 cm^–1^ (2.5–20 μm) of both amorphous and crystalline OCS ice and calculate the band strengths of all of its fundamental vibrational modes using the most recently measured refractive index and density of amorphous OCS ice.^8^ Then, we aim to aid deeper investigations into the chemical environment of interstellar OCS ice molecules by providing laboratory IR spectra of OCS in more chemically complex, tertiary ice mixtures with a focus on mixtures rich in CH_3_OH and CO, given their aforementioned plausible physicochemical links to OCS, and because interstellar CH_3_OH ice is thought to form primarily via CO hydrogenation after catastrophic CO freezeout,^21,22^ so it is expected and observationally supported^23,24^ that CO and CH_3_OH will be mixed in interstellar ices to some degree. Some of the investigated mixtures also include H_2_S, a suggested chemical precursor of OCS,^17,19^ and H_2_O, the most abundant observable interstellar ice.^25^ We also provide updated IR spectra of binary OCS ice mixtures using modern ice codeposition methodologies that result in more accurate and controlled ice mixing ratios.^8^ We report the peak positions, fwhms, and relative integrated absorbances of the OCS ν_3_ modes observed in these mixtures at temperatures spanning from 15 to 100 K to facilitate future analyses of astronomically observed OCS ice profiles. The spectral data are made publicly available on the Leiden Ice Database (LIDA).^26^ Finally, we compare our updated database of OCS ice data to previous OCS ice observations to determine which of our mixtures are most likely to be analogous to the chemical environment of the OCS in interstellar ices and calculate the apparent band strengths of the OCS ν_3_ mode in these mixtures. This set of experimental data can be used by the community to improve the understanding of the abundance, physicochemical environment, and evolutionary history of interstellar OCS ice in an era of copious new ice observations in environments like cold dark clouds, the faintest and youngest low-mass protostars, and extragalactic star-forming regions that have been enabled by the recently launched *James Webb* Space Telescope.

## Methods

### Experimental Setup

All of the data presented in this work were collected using the Infrared Absorption System for Ice Spectroscopy (IRASIS) at the Leiden Laboratory for Astrophysics. A schematic of the setup is presented by Rachid et al.,^27^ and subsequent upgrades are described by Slavicinska et al.^28^ To briefly summarize, the setup consists of an ultrahigh vacuum chamber (ultimate pressure <1 × 10^–9^ mbar) containing a KBr substrate that can be cooled down to 15 K with a closed-cycle He cryostat. Three independent dosing lines with independent gas reservoirs, leak valves, and chamber inlets located at the bottom of the chamber enable the background vapor deposition of ices on the cryo-cooled substrate. The deposition of these ices can be monitored on both sides of the substrate via laser interferometry with two 633 nm HeNe laser beams positioned at 45° relative to the substrate normal as well as via transmission IR spectroscopy with an FTIR beam positioned at 0° relative to the substrate normal. Ices are typically deposited to thicknesses on the order of hundreds to thousands of monolayers (depending on what thickness is needed to achieve satisfactory S/N of the ice feature of interest). The sample temperature can be kept constant or changed at a set rate via proportional-integral-derivative control. Gaseous species present in the chamber during deposition or warming are monitored with a quadrupole mass spectrometer (QMS).

In the experiments presented in this work, deposition pressures ranged from the order of 1 × 10^–8^ mbar in the case of the pure OCS ices to the order of 1 × 10^–6^ mbar in the case of the most diluted OCS ice mixtures. Deposition time scales ranged from ∼20 to 50 min. The IR spectra presented in this work were collected from 4000 to 500 cm^–1^ (2.5–20 μm) with a spectral resolution of 0.5 cm^–1^. Each spectrum consists of 128 averaged scans collected over a period of ∼3.5 min. During the warm-ups, the substrate was heated at a rate of 25 K h^–1^, resulting in ∼1.5 K intervals between the spectra collected during warm-up.

The liquids and gases used in this work include carbonyl sulfide (Linde Gas, ≥99.9%), methanol (Sigma-Aldrich, ≥99.9%), carbon monoxide (Linde Gas, ≥99.997%), water (Milli-Q, Type I), and hydrogen sulfide (Linde Gas, ≥99.5%).

### Mixing Ratio Calibration

This work uses similar methods to those described by Yarnall and Hudson^8^ and Slavicinska et al.^28^ to create binary and tertiary ice mixtures with specific ice component ratios. The position of each leak valve is independently calibrated to output the desired deposition rate of each ice component. This is achieved by first depositing each species of interest in pure form at 15 K and simultaneously monitoring the laser interference pattern and the QMS signal of a selected mass peak of the species of interest throughout the deposition. The period of the laser interference pattern *T* during deposition can be converted to an ice column density growth rate *dN/dt* via the following equation

1 $$\frac{\mathrm{dN}}{\mathrm{dt}}=\frac{\lambda }{2\sqrt{n^{2}\;\mathrm{si}n^{2}\;\theta }}\times \frac{\rho N_{A}}{\mathrm{MT}}$$

where λ is the wavelength of the laser, *n* is the ice refractive index, θ is the laser angle of incidence, ρ is the ice density, *N*_A_ is Avogadro’s number, and *M* is the ice molar mass. The literature *n* and ρ values used to obtain the ice column density growth rates of the species investigated in this work are presented in Table 1. The total errors on these growth rates stem from the uncertainties of the literature *n* and ρ values used, estimated here to be on the order of ∼5 and 15%, respectively, based on typical variations seen in these values between measurements made in different laboratories and with different setup configurations.^29–31^ The variations in ice densities measured in different laboratories are often primarily due to different deposition methods resulting in different ice porosities in the case of amorphous ices,^30^ where background deposition (the method employed in this work) typically produces more porous ice than directional deposition (the method employed by, e.g., Yarnall and Hudson^8^), although the magnitude of variation depends on the particular species. Additionally, there is a ±5° uncertainty of the laser angle of incidence, which corresponds to an error of ∼4% in the ice growth rates. This propagates to a total uncertainty of the ice growth rate of 16%.

**Table 1:** Literature Ice Refractive Index and Density Values of Pure Ices Used to Calculate the Ice Column Density Growth Rates in This Work

species	*n*	ρ (g cm^–3^)	refs
OCS	1.436	1.248	^8^
CH_3_OH	1.257	0.636	^32^
CO	1.297	0.870	^31^
H_2_O	1.234	0.719	^8^
H_2_S	1.407	0.944	^8^

A calibration curve is then generated by repeating pure ice deposition with at least five different growth rates and finding the best-fit correlation between the calculated ice column density growth rates and the average QMS signal of a selected mass peak throughout the deposition. For all of the investigated molecules, the correlation was well fit using a linear equation, with *R* ^2^ values ranging from 0.995 to 0.9998. Each leak valve can then be calibrated to a desired deposition rate of a specific species by filling the gas reservoir connected to that leak valve with a specific pressure of the gas or vapor of the species and then adjusting the leak valve until the QMS signal that corresponds to the desired ice column density growth rate in the calibration curve is reached. The exact deposition rate is ascertained by performing multiple laser interference control measurements of pure ice deposition rates using the calibrated leak valve position. Five of such measurements typically have a standard deviation of 0.3–3%, and their average varies by 2–5% from the desired growth rate used to calculate the corresponding QMS signal on the calibration curve. However, these experimental errors are negligible compared to the uncertainty on the calculated ice growth rate caused by the estimated uncertainty of the literature ρ value.

After this calibration is complete for all of the desired ice components, an ice mixture with specific component ratios can be deposited. First, each gas reservoir is filled with the same pressure of the pure gas or vapor used during the leak valve calibration. During this step, deposition of the gases or vapors into the chamber via the opened leak valves is prevented via shut valves preceding the leak valves. Once all of the gas reservoirs have been filled, all of the shut valves are opened to begin simultaneous deposition. As mentioned by Yarnall and Hudson,^8^ this method assumes that the ice column density growth rates during codeposition remain the same as during the deposition of each component individually.

### Apparent Band Strengths

For pure OCS ice, apparent band strengths were calculated by depositing six pure OCS ices at five different deposition rates (one deposition rate was repeated to check for consistency), with rates ranging from ∼0.2 to 2 μm h^–1^. Throughout each deposition, transmission IR spectra of the growing ice sample were continuously collected. The changes in the integrated optical depths of the OCS features of interest over time *d* ∫*τ dν*/*dt* were determined by finding the best linear fits to the integrated optical depths over time. High *R* ^2^ values of these fits in the case of the ν_3_ feature (≥0.9996) indicate very uniform ice growth rates throughout all of the depositions. The corresponding ice column density growth rate for each of these measurements was then determined by using eq 1 to convert the period of the laser interference pattern generated during each deposition to *dN/dt*.

The best linear fits to the six independent *d* ∫*τ dν*/*dt* versus *dN/dt* measurements, plotted in time-derivative Beer’s law plots, were then determined via the York method for fitting data with errors on both *X* and *Y* variables.^33^ The slopes of these fits are reported as the apparent band strengths, *A*′, of the pure OCS bands. Using the time derivatives of the ice column densities and integrated optical depths rather than the absolute values of these variables ensures that any residues from previous experiments present on the substrate do not contribute to the calculated ice column densities. It also eliminates errors caused by human imprecision in stopping the deposition at a specific time or ice thickness. The error of the slope calculated by the York method yields *A*′ uncertainties ∼10%, with the dominant source of uncertainty being the uncertainty of the ρ value used in eq 1.

For the OCS ice mixtures, apparent band strengths were determined only for the ν_3_ mode, and they were calculated via a different method than the pure apparent band strengths, as the ice column density growth rates could not be determined from the laser interference patterns since the *n* and ρ values of the ice mixtures are not known. Instead, the leak valve used to deposit OCS was calibrated to correspond to a specific OCS ice growth rate, and five pure OCS ice depositions were performed using this leak valve position. The same leak valve position was then used to dose OCS during the deposition of the ice mixture of interest. The apparent band strength of the OCS feature in each mixture was subsequently calculated using the equation

2 $$A_{mix}^{\prime }=A_{pure}^{\prime }\times \frac{d\;\int \tau_{mix}\;d\nu /\mathrm{dt}}{d\;\int \tau_{pure,\mathrm{avg}}\;d\nu /\mathrm{dt}}$$

where *d* ∫τ_mix_ *dν*/*dt* is the change in the integrated optical depth of the OCS ν_3_ feature during the deposition of each mixture of interest, *d* ∫τ_pure,avg_ *dν*/*dt* is the average change in the integrated optical depth of the OCS ν_3_ feature during the five pure OCS depositions (standard deviation = 0.8%), and *A*_pure_^′^ is the apparent band strength of the ν_3_ mode of pure OCS.

## Results

### Pure OCS Ice

The IR spectrum of pure OCS ice in both amorphous and crystalline forms is presented in Figure 1. The ν_3_ (asymmetric stretching) mode around 5 μm is by far the strongest feature. Its profile in both the amorphous and crystalline forms is highly asymmetric and distinctly broad, which has been suggested to be caused by the isotopic shifts of and disruption of molecular coupling in the matrix by OC^33^S and OC^34^S.^34^ The other fundamental features, the ν_1_ (symmetric stretching) and ν_2_ (bending) modes, are significantly weaker than the ν_3_ modes in both the amorphous and crystalline ices. Several very weak combination and overtone bands are also present in the investigated spectral range. The peak positions of all of the assigned modes are listed in Table 2. A small peak at 2340 cm^–1^, assigned to CO_2_, indicates a slight degree of atmospheric contamination. Using the band strength of the asymmetric stretching mode of pure amorphous CO_2_,^35^ this contamination is estimated to be on the order of ∼0.2% with respect to OCS. A table containing the peak position, fwhm, and relative integrated absorbance of the ν_3_ mode in pure OCS ice throughout its warm-up from 15 to 83 K is provided in the Supporting Information.

**Table 2:** Positions and Assignments of IR Absorption Features in Pure OCS Ice Spectra Measured at 15 K (Amorphous) and 65 K (Crystalline)

assignment^a^	peak position 15 K		peak position 65 K	
	(cm^–1^)	(μm)	(cm^–1^)	(μm)
2ν_2_+ν_3_	3056.3	3.272	3058.7	3.269
ν_1_+ν_3_	2881.4	3.471	2885.2	3.466
CO_2_ contam.	2339.2	4.275	2334.7	4.283
4ν_2_?			2092.4	4.779
ν_3_ (asymm. str.)	2033.7	4.917	2003.7	4.991
ν_3_^18^OCS			1998.1	5.005
ν_3_ O^13^CS	1984.3	5.040	1982.9	5.043
ν_1_+2ν_2_ ^b^	1883.2	5.310	1885.2	5.304
			1880.2	5.319
2ν_1_	1707.7	5.856	1707.1	5.858
2ν_2_	1042.0	9.597	1042.1	9.596
ν_1_ (symm. str.)	857.3	11.665	857.5	11.662
ν_2_ (bend)	513.1	19.489	518.9	19.272

a Based on assignments from Verderame and Nixon.^34^

b Feature is split in the crystalline spectrum.

**Figure 1:**
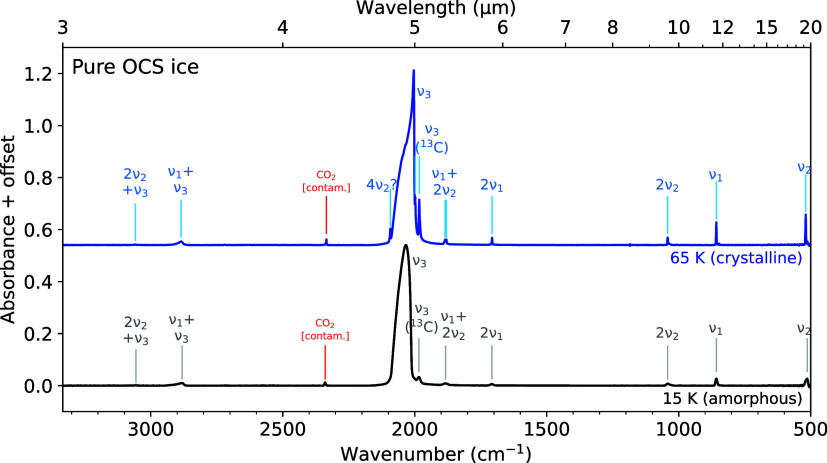
Infrared spectra of amorphous (black trace) and crystalline (blue trace) pure OCS ices measured at 15 and 65 K, respectively. Both ices were deposited at 15 K; the crystalline ice was heated from 15 to 65 K at a rate of 25 K hr^–1^. Assignments to the absorption features are labeled, and peak positions of these features are listed in Table 2. A small feature ∼2340 cm^–1^ assigned to CO_2_ ice indicates minor atmospheric contamination.

The apparent band strengths *A*′ of the three fundamental OCS modes in pure amorphous ice at 15 K were calculated using the method outlined in the Methods section. The resulting *A*′ values are presented in Table 3, and the time-derivative Beer’s law plots used to calculate these values are shown in Figure 2. Our *A*′ value for the ν_3_ mode, 1.20 × 10^–16^ cm molec^–1^, is exactly consistent with that reported for pure amorphous OCS ice at 10 K by Yarnall and Hudson.^8^ However, we note that our chosen integration range for this peak differs from that listed in Table 2 in Yarnall and Hudson,^8^ 2154–2070 cm^–1^, because their range excludes a majority of the absorption of this feature, leading us to conclude it likely includes a typo.

**Table 3:** Band Strengths of the Fundamental Vibrational Modes of OCS Ice, Both Pure and in Mixtures with CH_3_OH and CO in the Case of the ν_3_ Mode, at 15 K

feature	ice	ratio	peak position		*A*′	integration range
			(cm^–1^)	(μm)	(cm molec^–1^)	(cm^–1^)
ν_3_	OCS		2033.7	4.917	1.20 (±0.12) × 10^–16^	2197–1909
	OCS:CH_3_OH	1:20	2041.5	4.898	1.21 (±0.13) × 10^–16^	2106–1999
	OCS:CH_3_OH	1:40	2041.5	4.898	1.18 (±0.13) × 10^–16^	2106–1999
	OCS:CO:CH_3_OH	1:20:20	2040.8	4.900	1.19 (±0.13) × 10^–16^	2078–1999
	OCS:CO:CH_3_OH	1:40:20	2041.3	4.899	1.20 (±0.13) × 10^–16^	2078–1999
	OCS:CO:CH_3_OH	1:20:10	2041.2	4.899	1.21 (±0.13) × 10^–16^	2078–1999
	OCS:CO:CH_3_OH	1:40:10	2044.7	4.891	1.24 (±0.13) × 10^–16^	2078–1999
ν_1_	OCS		857.2	11.67	8.86 (±0.91) × 10^–19^	867–839
ν_2_	OCS		514.2	19.45	1.03 (±0.11) × 10^–18^	530.5–505.8

**Figure 2:**
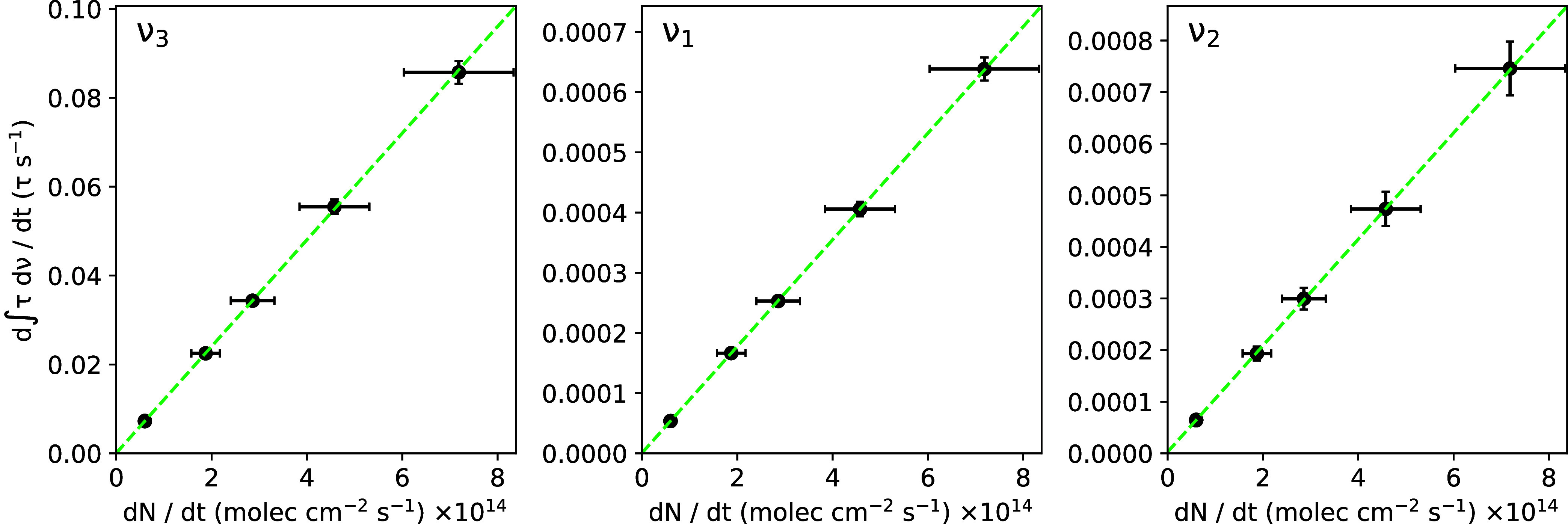
Time-derivative Beer’s law plots used to derive the apparent band strengths of the fundamental IR modes of amorphous OCS ice at 15 K. The black points denote the experimental data, with each point representing an individual ice deposition with a given deposition rate in units of column density growth per second, and the dashed green lines denote the best linear fit to the data calculated using the York method.

Our measured *A*′ values of the OCS ν_1_ and ν_2_ modes are both over 2 orders of magnitude lower than that of the ν_3_ mode. Given such low *A*′ values in combination with the relatively low abundance of OCS in interstellar ices as determined from the ν_3_ mode, we conclude that these bands are not likely to be practical in future observational studies and, therefore, exclude them from the analysis of the ice mixtures.

### OCS Ice Mixtures

The profiles of the OCS ν_3_ features at select temperatures in all of the ice mixtures investigated in this work are presented in Figure 3. In Figure 4, the extracted peak positions and fwhms of the OCS ν_3_ features from these experiments are overplotted, with the peak positions and fwhms extracted from Gaussian fits to the 4.9 μm feature observed toward massive protostars (or massive young stellar objects; MYSOs) by Boogert et al.^6^ (The binary OCS:CO mixtures and the 1:20:1 mixtures of the OCS:CO:H_2_S are excluded from this figure due to their poor fits to the observations and substructures with multiple peaks.) Tables containing the peak positions, fwhms, and relative integrated absorbances of the OCS ν_3_ mode in these ice mixtures throughout their warm-up from 15 to 100 K are provided in the Supporting Information.

**Figure 3:**
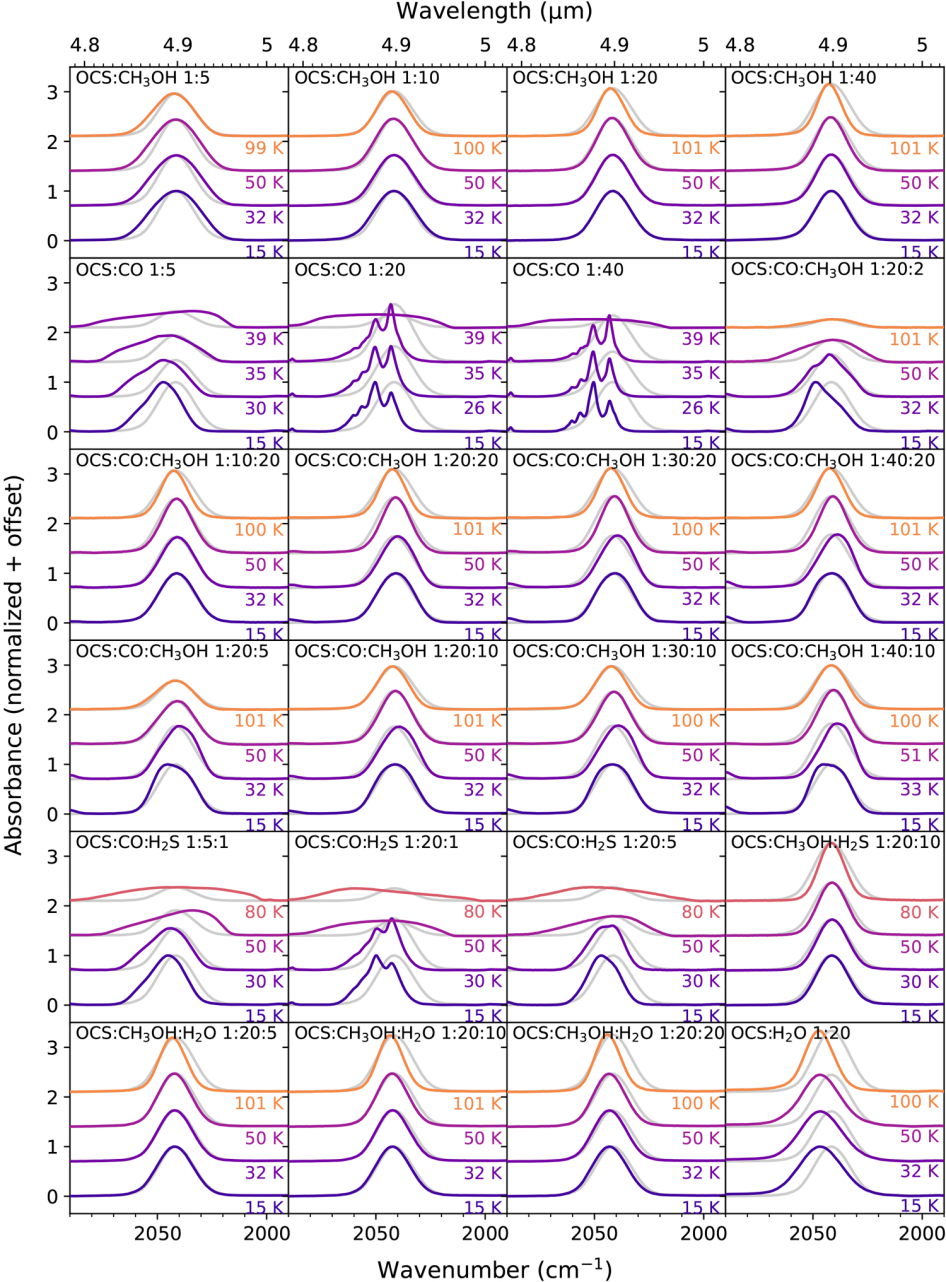
OCS ν_3_ bands at select temperatures in all of the investigated binary and tertiary OCS-containing ice mixtures. All of the spectra are normalized so that the peak absorbance of the ν_3_ mode at 15 K is equal to 1 in each set of spectra from a given mixture. The profile of the ν_3_ mode in the 1:20 ice mixture of the OCS:CH_3_OH at 15 K is scaled to and plotted with all of the peaks (in gray) to facilitate comparisons of peak positions and profiles between the different mixtures.

**Figure 4:**
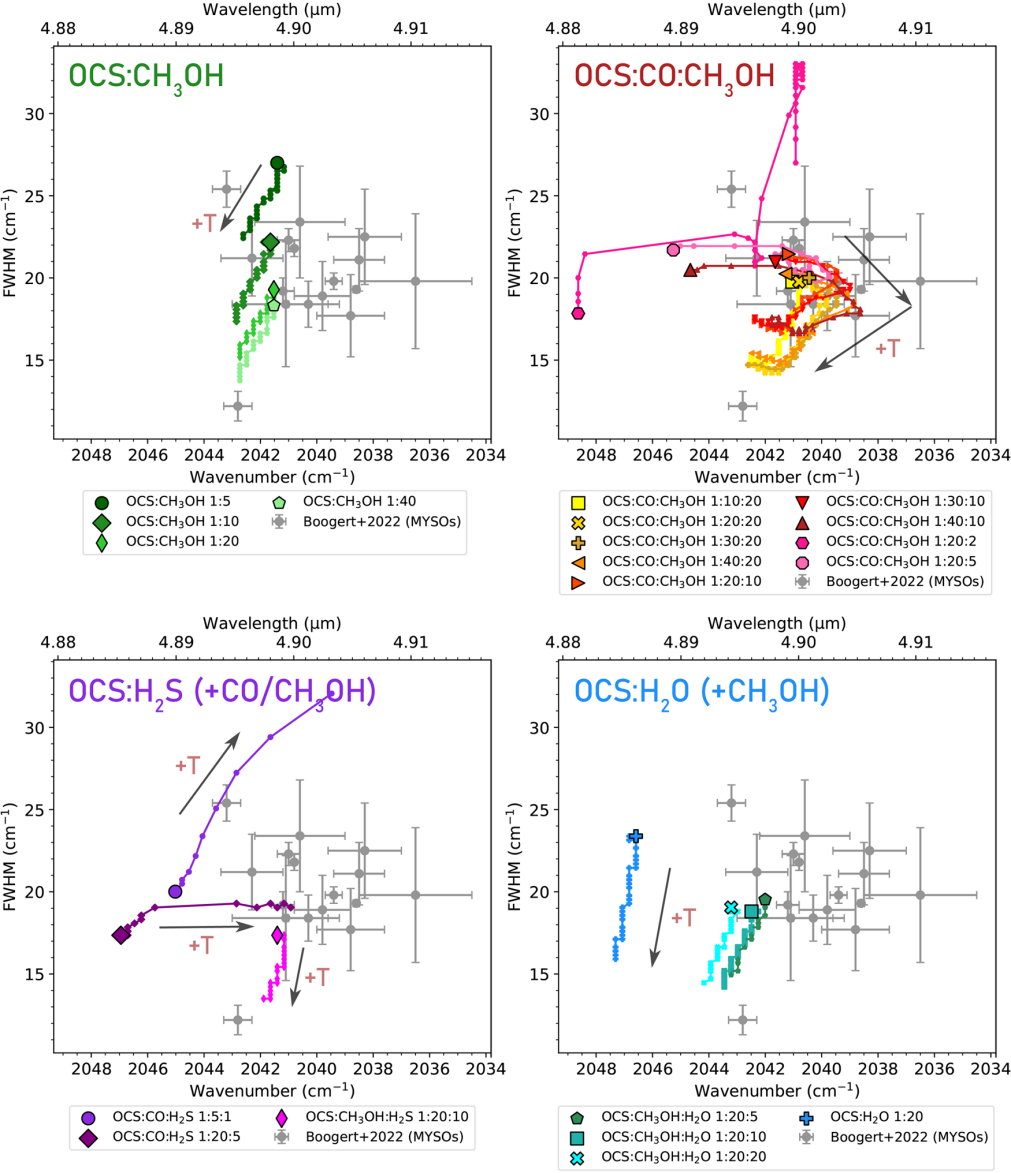
Peak positions and fwhms of the OCS ν_3_ mode in all of the investigated laboratory mixtures (color), excluding the binary OCS:CO mixtures and the 1:20:1 mixture. The large outlined symbols denote mixtures at 15 K, while the traces and smaller symbols denote how the peak profile changes with increasing temperature. The black arrows indicate the direction of temperature increase. All mixtures are plotted up to 100 K except the OCS:CO:H_2_S mixtures, which are only plotted up to 37 K because above this temperature, the OCS ν_3_ feature in these mixtures becomes significantly broader than the peak in observations. The peak positions and fwhms of the Gaussian profiles fit to the 4.9 μm band observed toward massive young stellar objects (MYSOs) by Boogert et al.^6^ are plotted in gray.

#### OCS:CH_3_OH Ice Mixtures

In all of the binary OCS:CH_3_OH mixtures, the OCS peak profile is symmetric and Gaussian-like, and its position is sensitive to temperature: it lies around ∼2041.5 cm^–1^ (4.898 μm) at 15 K and gradually blue-shifts with increasing temperature to ∼2042.7 cm^–1^ (4.895 μm) at 100 K in all of the investigated OCS:CH_3_OH mixing ratios. The fwhm, on the other hand, is sensitive to both the temperature and the OCS:CH_3_OH ratio. For example, the fwhm decreases from ∼23 to ∼19 cm^–1^ when the OCS:CH_3_OH concentration decreases from 1:10 to 1:20 at 15 K, but a similar decrease in the fwhm also occurs during the warm-up of the OCS:CH_3_OH 1:10 mixture. However, differences between the fwhms of the 1:20 and 1:40 mixtures of the OCS:CH_3_OH are much smaller. Based on this trend, we expect that increasing the dilution factor of the OCS:CH_3_OH beyond 1:40 would have very small effects on the OCS peak profile.

Notably, while several of the peak profiles observed by Boogert et al.^6^ can be well fit with our OCS:CH_3_OH mixtures, many other observed peak positions are too red-shifted (by 0.001–0.01 μm) to be sufficiently fit with them.

Given the similarity of these spectra to some of the observations, we derived apparent band strengths of the ν_3_ band in the 1:20 and 1:40 mixtures of the OCS:CH_3_OH using the procedure described in the Methods section and report these values in Table 3. Within our uncertainties, these *A*′ values are consistent with the *A*′ value of pure OCS.

#### OCS:CO Ice Mixtures

Mixing OCS with oxazoline (AgCl) and CO results in an OCS peak blue shift of ∼5–10 cm^–1^ with respect to the OCS peak positions in the OCS:CH_3_OH mixtures. Such a peak position is too blue-shifted to fit observations. Furthermore, at the higher dilution factors (OCS:CO 1:20 and 1:40), the OCS feature splits into four blended but distinct peaks (Figure 3). Similar splitting is observed in OCS:Ar and OCS:N_2_ 1:200 mixtures, where it has been attributed to isolated and aggregated OCS molecules within the Ar or N_2_ matrices,^34^ as the multiple peaks eventually disappear at even higher dilution factors. These split peak profiles do not match the observed Gaussian-like profile of interstellar OCS ice. Between 30 and 40 K, the multipeak structures transform into very broad, asymmetric features similar to the peak structure of pure OCS ice because the CO ice desorbs between these temperatures, effectively distilling the ice and leaving behind nearly pure solid OCS.

#### OCS:CO:CH_3_OH Mixtures

Most of the investigated tertiary OCS:CO:CH_3_OH mixtures have similar OCS profiles to the OCS:CH_3_OH mixtures but with two notable differences. First, the profiles of the OCS:CO:CH_3_OH mixtures are slightly asymmetric at lower temperatures. The effect is most pronounced at 15 K and in the mixtures with the lowest CH_3_OH concentrations (e.g., see the OCS:CO:CH_3_OH 1:20:2 peak in Figure 3) but is still observed in the spectra up to ∼40 K, before a significant portion of the deposited CO begins to desorb. Second, the OCS peak positions in these mixtures tend to be slightly blue-shifted relative to the OCS:CH_3_OH mixtures at 15 K, but as the temperatures increase to ∼30–35 K, the peaks shift toward longer wavelengths. In the OCS:CO:CH_3_OH 1:40:10 mixture, a maximum red shift is achieved at 35 K, with a peak position of 2038.6 cm^–1^ (4.905 μm). Such red-shifted profiles provide substantially better fits to many of the red-shifted observations.

It is important to note that these experiments demonstrate how even a small concentration of CH_3_OH in the matrix has a pronounced effect on the OCS peak profile—i.e., the mixture does not have to be CH_3_OH-dominated for the OCS peak to have a profile similar to that in the OCS:CH_3_OH mixtures. For example, in the CO-dominated OCS:CO:CH_3_OH 1:20:5 mixture, the OCS peak profile looks much more similar to the peak in the OCS:CH_3_OH 1:5 mixture than in the OCS:CO 1:20 mixture (see Figure 3).

Given the match in the red-shifted peak position of several of the OCS:CO:CH_3_OH mixtures to the MYSO observations in Boogert et al., we calculated the apparent band strengths of the OCS ν_3_ mode in the 1:20:20, 1:40:20, 1:20:10, and 1:40:10 mixtures using the procedure outlined in the Methods section and report these values in Table 3. As was the case for the OCS:CH_3_OH mixtures, these *A*′ values are consistent with the *A*′ value of pure OCS within our reported uncertainties.

#### OCS:CO:H_2_S and OCS:CH_3_OH:H_2_S Mixtures

The three investigated tertiary OCS:CO:H_2_S mixtures have significantly blue-shifted and markedly asymmetric OCS peaks at 15 K. As they are heated to 30–40 K, their peaks red-shift toward the observed values but remain quite asymmetric, making them poor fits to the observations despite the apparent experimental overlap with the observational data in Figure 4. At higher temperatures, much of the deposited CO desorbs and the OCS peaks become much broader than the observations as the OCS ice is effectively distilled. Further OCS distillation occurs in these mixtures between 70 and 80 K when H_2_S desorbs.

The OCS peak profile of the OCS:CH_3_OH:H_2_S 1:20:10 mixture is nearly identical to that of the most dilute OCS:CH_3_OH mixtures. Therefore, the OCS peak profile is not sensitive to the presence of H_2_S in CH_3_OH-dominated matrices.

#### OCS:H_2_O and OCS:CH_3_OH:H_2_O Mixtures

The OCS profile in the investigated H_2_O-containing mixtures is symmetric and Gaussian-like, similar to that of the binary CH_3_OH mixtures. The binary OCS:H_2_O 1:20 mixture is far too blue-shifted (by at least ∼5 cm^–1^) to fit any of the observations, as noted previously by Palumbo et al.^10^ However, the tertiary mixtures of OCS:CH_3_OH:H_2_O explored here, even those with equivalent concentrations of CH_3_OH and H_2_O like the 1:20:20 mixture, have OCS peak profiles and positions that are very similar to those of the binary OCS:CH_3_OH mixtures. They are also slightly blue-shifted but not by enough to lie outside of the observed OCS peak profile range considering errors. These results demonstrate again that the presence of CH_3_OH in the ice matrix has a strong effect on the OCS peak profile, even if it is not the only dominant matrix species.

## Astronomical Implications

With the spectra collected in this work, we show that many of the observed OCS ν_3_ peak positions that were too red-shifted to be fit with any previously available nonirradiated laboratory ice mixtures can now actually be fit quite well with tertiary OCS:CO:CH_3_OH mixtures. In the bottom two panels of Figure 5, we demonstrate how OCS:CO:CH_3_OH mixtures provide good fits to the OCS ice features observed toward W33A and G029.8620–00.0444 (hereafter G029.8620), two embedded massive protostars observed via the NASA InfraRed Telescope Facility (IRTF).^6^ The massive protostar G029.8620 in particular has the highest quality of the IRTF sample and is shifted significantly to longer wavelengths with respect to binary OCS:CH_3_OH mixtures. This is the case even after the correction for a Doppler velocity shift of +100 km s^–1^ with respect to the Local Standard of Rest^36^ and the motion of the Earth around the Sun at the date of the IRTF observations (this correction was not applied in Boogert et al.^6^).

**Figure 5:**
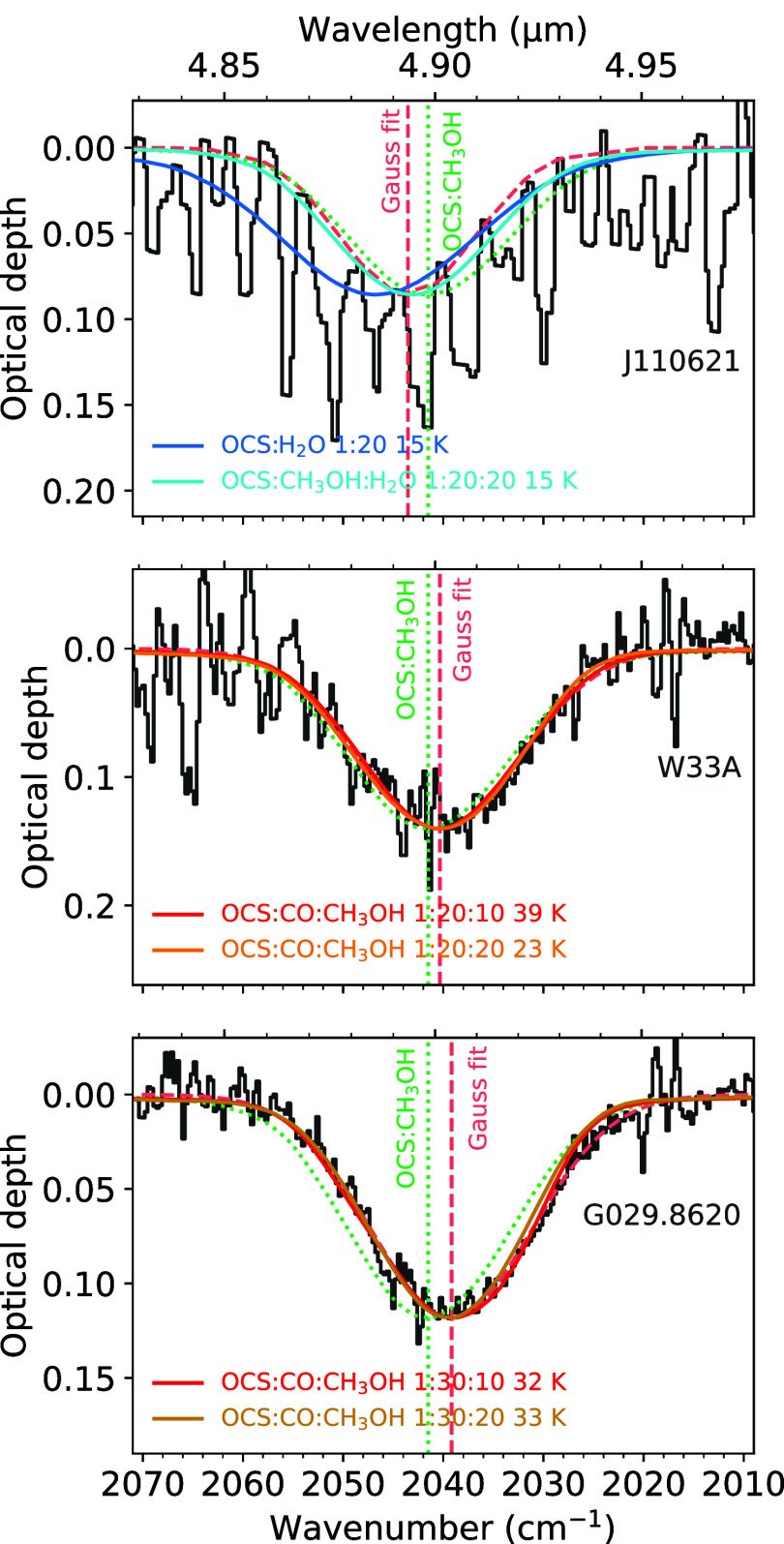
OCS ν_3_ mode observed toward three icy sightlines (gray traces) overplotted with select laboratory ice mixtures from this work (colored traces). A Gaussian fit to each observed feature (dashed coral trace) and the laboratory OCS:CH_3_OH 1:20 15 K spectrum (dotted green trace), along with vertical lines marking their respective peak positions, are also overplotted to serve as points of reference and to facilitate visual comparisons between the different sources and laboratory data. The observational data include the dense core in front of a background star J110621 (top),^7^ the massive protostar W33A (middle),^6^ and the massive protostar G029.8620 (bottom).^6^ CO gas lines were removed from the MYSO spectra (see Boogert et al.^6^).

These laboratory spectra therefore provide an alternative explanation to that presented by Boogert et al.^6^ for the observed OCS peak positions—namely, that solid-state interstellar OCS may be intimately mixed with both CO and CH_3_OH ice. This result is one of many that supports a close chemical relationship between CO and CH_3_OH^21–24^ and is consistent with the hypothesis that CO is a chemical precursor of OCS.^16^ Unfortunately, the analysis of more precise physicochemical parameters like the OCS:CO:CH_3_OH ratio and temperature of the ice matrix surrounding OCS cannot be reliably determined by analyzing the OCS ν_3_ peak profile due to the degenerate effects of mixing ratios and temperatures on both the OCS peak position and fwhm in the OCS:CO:CH_3_OH laboratory mixtures.

Conversely, our data do not provide conclusive evidence for OCS mixing with H_2_S, another suggested OCS precursor,^17,19^ in interstellar ices, although they do not refute the possibility, either. The 1:20:10 mixture of the aqueous OCS:CH_3_OH:H_2_S demonstrates that the presence of H_2_S in a CH_3_OH-dominated matrix does not have a strong effect on the OCS peak profile, even at relatively high (10:1) H_2_S:OCS ratios. Significantly higher concentrations of H_2_S are unlikely given that currently available H_2_S ice upper limits in the literature result in H_2_S:OCS ratio upper limits of ≲4.^1,7^ The asymmetric profiles of the OCS bands in the OCS:CO:H_2_S mixtures investigated here do not match the more symmetric profiles of the OCS features reported in irradiated laboratory CO:H_2_S ice mixtures,^19,20^ which may be due to the presence of several other irradiation products in the latter ices. It is possible that including CH_3_OH in the nonirradiated OCS:CO:H_2_S mixtures could result in more symmetric OCS features that would better match observations, but any changes in the OCS peak profile due to H_2_S in such mixtures are likely to be small (given the tight constraints on H_2_S ice abundances from the current upper limits) and perhaps also degenerate with other physicochemical effects.

Finally, we include in Figure 5 one of the first observations of OCS ice toward a dense core in front of a background star, J110621, observed by the *James Webb* Space Telescope (JWST).^7^ Although the feature is contaminated by CO gas absorption lines from the photosphere of the background star, a peak profile can be roughly extracted by fitting to the data points between the absorption lines, resulting in an extracted peak position that is blue-shifted relative to the OCS:CH_3_OH 1:20 position. The laboratory data from this work that provide the best match to such a profile are the 1:20:20 OCS:CH_3_OH:H_2_O 1:20:20 mixture. In their analysis of this source, McClure et al.^7^ found that the local cloud density may still be too low to support catastrophic CO freezeout, and that some portion of the detected CH_3_OH ice may be mixed with H_2_O, possibly due to early CH_3_OH formation via a pathway like CH_4_+OH in a H_2_O-rich environment.^37^ The blue shift in the OCS peak position toward this source could be considered evidence for early formation of both the OCS and CH_3_OH in a more H_2_O-rich ice phase, although this evidence remains very tenuous given that the overlapping photospheric contamination may create distortions in the observed OCS profile, leading to higher uncertainties in the extracted peak position.

Given the spectroscopic degeneracies, we do not claim that our laboratory spectra provide unique fits to the observed OCS ice observations. Therefore, our fits do not strictly preclude other chemical environments, like those investigated by Ferrante et al.^19^ and Garozzo et al.,^20^ providing equally satisfactory fits. Our fits do provide evidence supporting plausible chemical environments of OCS ice comprising only simple ice species that are securely and abundantly observed in dense star-forming regions.

## Conclusions

This work provides the astrochemical community with a library of new laboratory IR transmission spectra of OCS-containing ice mixtures to aid the analysis of observations of interstellar ices. Apparent band strengths, peak positions, and fwhms of the OCS asymmetric stretching mode (ν_3_) in these mixtures are reported. The main conclusions from this work can be summarized as follows:

- Tertiary laboratory mixtures of OCS:CO:CH_3_OH provide the best fits to most of the OCS ice bands currently observed toward the ice envelopes of massive protostars, supporting the previously hypothesized link between these species. The match also supports the late OCS formation scenario, in which most of the observed OCS ice forms in the densest, coldest regions of ice clouds and envelopes where CO freezeout occurs.
- CH_3_OH has a strong effect on the profile of the OCS band even when it is not the dominant ice component, and OCS features observed toward several massive protostars can be fit well with OCS:CO:CH_3_OH laboratory ice mixtures whose dominant component is CO rather than CH_3_OH.
- Specific conclusions regarding local OCS concentration or OCS ice temperature based on the OCS peak profile are hindered by the degenerate effects of these parameters. Taking into account previous work, this degeneracy also applies to proton-irradiated ices containing H_2_S, CO, and H_2_O.^19^
- The apparent band strength of OCS in OCS:CH_3_OH and OCS:CO:CH_3_OH mixtures is consistent with that of pure OCS ice within our reported uncertainties. We recommend that a value of 1.2 × 10^–16^ cm molec^–1^ is used to calculate OCS column densities in interstellar ices.
- Our data neither support nor refute possible chemical links between H_2_S and OCS, as the OCS:CO:H_2_S mixtures collected as part of this work have asymmetric OCS profiles that do not match any observations, but the investigated OCS:CH_3_OH:H_2_S mixture has a similar profile to binary OCS:CH_3_OH mixtures that fit some of the observed OCS peaks well.
- While OCS:H_2_O mixtures are too blue-shifted to fit any current OCS observations, OCS:CH_3_OH:H_2_O mixtures may provide a good fit to an OCS feature observed toward a background star behind a dense cloud, suggesting that some OCS may form earlier in a phase of the ice that is still relatively H_2_O-rich. However, this conclusion remains tenuous due to spectral contamination by photospheric CO gas absorptions complicating the OCS profile extraction toward this source.
- Given the weak apparent band strengths of the OCS symmetric stretching (ν_1_) and bending (ν_2_) modes and the relatively low abundance of OCS ice in dense star-forming regions, the very strong OCS asymmetric stretching (ν_3_) mode remains the only likely detectable feature of this molecule in interstellar ices in the near future.

These laboratory spectra are made available for public use and enjoyment on the Leiden Ice Database (LIDA).^26^

## Supplementary Material



## Notes

Tables containing the peak positions, fwhms, and relative integrated absorbances of the OCS ν_3_ mode in both pure and mixed OCS ice at selected temperatures spanning 15–100 K (PDF)
