# Supplementary material for: Laboratory Infrared Spectra and Band Strengths of Carbonyl Sulfide (OCS) in CH3OH- and CO-Rich Ice Mixtures for Analyzing Interstellar Ice Observations
Source: ACS Earth Space Chem. 2025 Jul 22;9(8):2148–58. doi: 10.1021/acsearthspacechem.5c00134 (PMC12376183; doi:10.1021/acsearthspacechem.5c00134)
Supplement: Supplementary file 1 [file sp5c00134_si_001.pdf]

Supporting information: Laboratory infrared spectra and band strengths of carbonyl sulfide (OCS) in CH_3OH- and CO-rich ice mixtures for analyzing interstellar ice observations

Katerina Slavicinska,[*,†,‡] Charlotte Coone,[†] Bryce Benz,[†] Harold Linnartz,[†] A. C. Adwin Boogert,[¶] and Ko-Ju Chuang[†]

†*Laboratory for Astrophysics, Leiden Observatory, Leiden University, P.O. Box 9513, NL 2300 RA Leiden, The Netherlands.*

‡*Leiden Observatory, Leiden University, P.O. Box 9513, NL 2300 RA Leiden, The Netherlands.*

¶*Institute for Astronomy, University of Hawai'i at Manoa, 2680 Woodlawn Drive, Honolulu, HI 96822, USA.*

E-mail: slavicinska@strw.leidenuniv.nl

OCS ν_3 peak positions, FWHMs, and relative integrated absorbances

This appendix presents the peak positions, FWHMs, and relative integrated absorbances (with respect to the integrated absorbance of each mixture at 15 K) of the OCS ν_3 feature in all OCS-bearing ice mixtures investigated in this work. The uncertainty of the peak positions and FWHMs is 0.5 cm^{-1}.

Table S1: Peak positions, FWHMs, and relative integrated absorbances (with respect to the integrated absorbance of each mixture at 15 K) of the OCS $\nu3$ feature in pure and binary OCS ices at various temperatures.

Matrix	T (K)	Peak cm^{-1}	Peak μm	FWHM cm^{-1}	FWHM μm	Rel. integ. abs. (w.r.t. 15 K)
OCS*	15	2034.1	4.916	55.9	0.134	1.00
	50	2029.7	4.927	53.5	0.128	1.03
	65	2003.7	4.991	46.3	0.113	1.07
	83	2003.4	4.991	27.5	0.068	1.13
OCS:CH_3OH 1:5	15	2041.4	4.899	27.0	0.065	1.00
	52	2041.6	4.898	25.3	0.061	0.99
	66	2042.1	4.897	24.3	0.058	0.98
	80	2042.4	4.896	23.6	0.057	0.97
	95	2042.6	4.896	22.7	0.054	0.85
OCS:CH_3OH 1:10	15	2041.6	4.898	22.2	0.053	1.00
	50	2042.1	4.897	20.5	0.049	0.99
	60	2042.4	4.896	19.8	0.047	0.98
	71	2042.6	4.896	19.0	0.046	0.98
	81	2042.8	4.895	18.3	0.044	0.97
	99	2042.8	4.895	17.4	0.042	0.76
OCS:CH_3OH 1:20	15	2041.5	4.898	19.3	0.046	1.00
	48	2042	4.897	17.8	0.043	1.00
	59	2042.2	4.897	17.4	0.042	0.99
	69	2042.5	4.896	16.6	0.040	0.99
	80	2042.7	4.895	15.9	0.038	0.98
	99	2042.7	4.895	15.2	0.036	0.82
OCS:CH_3OH 1:40	15	2041.5	4.898	18.3	0.044	1.00
	48	2042	4.897	16.6	0.040	1.00
	60	2042.2	4.897	16.2	0.039	0.99
	71	2042.5	4.896	15.4	0.037	0.98
	81	2042.7	4.895	14.7	0.035	0.97
	99	2042.7	4.895	13.7	0.033	0.85
OCS:CO 1:5*	15	2047.3	4.884	19.8	0.047	1.00
	32	2047.6	4.884	30.1	0.072	0.92
	35	2043.7	4.893	35.7	0.085	0.90
OCS:CO 1:20*	15	2050.2	4.878	14.0	0.033	1.00
	26	2043.2	4.894	14.7	0.035	1.01
	30	2043.2	4.894	12.8	0.030	1.02
	35	2043.2	4.894	12.3	0.029	1.03
OCS:CO 1:40*	15	2050.7	4.876	5.3	0.013	1.00
	31	2043	4.895	5.1	0.012	1.00
	35	2043	4.895	4.8	0.012	0.97

* indicates that the OCS ν_3 mode in the marked ice has a strongly asymmetric or split (i.e., non-Gaussian) peak profile.

Table S2: Peak positions, FWHMs, and relative integrated absorbances (with respect to the integrated absorbance of each mixture at 15 K) of the OCS $\nu3$ feature in select tertiary OCS:CO:CH_3OH mixtures at various temperatures.

Matrix	T (K)	Peak cm^{-1}	Peak μm	FWHM cm^{-1}	FWHM μm	Rel. integ. abs. (w.r.t. 15 K)
OCS:CO:CH_3OH 1:20:2*	15	2048.6	4.881	17.8	0.043	1.00
	25	2043.1	4.895	22.7	0.054	0.98
	41	2041.2	4.899	29.9	0.072	0.79
	50	2040.9	4.900	31.8	0.076	0.78
	71	2040.9	4.800	32.3	0.077	0.78
	90	2040.9	4.900	31.6	0.076	0.52
OCS:CO:CH_3OH 1:10:20	15	2041	4.899	19.8	0.047	1.00
	36	2040.8	4.900	18.6	0.045	1.00
	50	2041.3	4.899	17.1	0.041	0.98
	60	2041.5	4.898	15.9	0.038	0.98
	70	2041.8	4.898	15.2	0.036	0.98
	84	2042.2	4.897	14.7	0.035	0.97
	94	2042.5	4.896	15.2	0.036	0.90
OCS:CO:CH_3OH 1:20:20	15	2040.8	4.900	19.8	0.047	1.00
	33	2039.8	4.902	18.3	0.044	0.99
	47	2040.8	4.900	16.2	0.039	0.95
	60	2041.3	4.899	14.7	0.035	0.94
	72	2041.8	4.898	14.5	0.035	0.93
	90	2042.5	4.896	14.7	0.035	0.93
OCS:CO:CH_3OH 1:30:20	15	2040.4	4.901	20.0	0.048	1.00
	32	2039.5	4.903	18.6	0.045	1.00
	42	2040.4	4.901	16.6	0.040	0.97
	51	2040.9	4.900	15.7	0.038	0.96
	59	2041.2	4.899	14.9	0.036	0.96
	65	2041.4	4.899	14.5	0.035	0.96
	72	2041.6	4.898	14.5	0.035	0.95
	83	2042.1	4.897	14.7	0.035	0.94
	93	2042.6	4.896	14.7	0.035	0.89
OCS:CO:CH_3OH 1:40:20	15	2041.3	4.899	20.2	0.049	1.00
	22	2040.6	4.901	20.2	0.049	1.01
	32	2038.9	4.905	18.6	0.045	1.02
	41	2040.6	4.901	16.6	0.040	0.95
	50	2040.8	4.900	15.9	0.038	0.95
	60	2041.3	4.899	15.2	0.036	0.95
	71	2041.5	4.898	14.9	0.036	0.95
	80	2041.8	4.897	15.2	0.036	0.93
	90	2042.2	4.897	15.2	0.036	0.93
	99	2042.5	4.896	15.4	0.037	0.86

* indicates that the OCS ν_3 mode in the marked ice has a strongly asymmetric or split (i.e., non-Gaussian) peak profile.

Table S3: Peak positions, FWHMs, and relative integrated absorbances (with respect to the integrated absorbance of each mixture at 15 K) of the OCS $\nu3$ feature in select tertiary OCS:CO:CH_3OH mixtures at various temperatures.

Matrix	T (K)	Peak cm^{-1}	Peak μm	FWHM cm^{-1}	FWHM μm	Rel. integ. abs. (w.r.t. 15 K)
OCS:CO:CH_3OH 1:20:5	15	2045.3	4.889	21.7	0.052	1.00
	22	2043.1	4.895	21.9	0.053	1.00
	35	2039.7	4.903	20.0	0.048	1.01
	50	2040.7	4.900	20.2	0.049	0.85
	60	2040.7	4.900	20.0	0.048	0.84
	71	2040.9	4.900	20.2	0.049	0.83
	80	2041.2	4.899	20.5	0.049	0.83
	90	2041.4	4.899	21.5	0.051	0.78
	96	2041.6	4.898	21.2	0.051	0.68
OCS:CO:CH_3OH 1:20:10	15	2041.2	4.899	21.5	0.051	1.00
	20	2040.7	4.900	21.2	0.051	1.00
	30	2039.5	4.903	20.0	0.048	1.00
	38	2040.2	4.901	19.0	0.046	0.95
	45	2040.7	4.900	18.3	0.044	0.94
	56	2041.2	4.899	17.1	0.041	0.93
	65	2041.4	4.899	16.9	0.040	0.93
	75	2041.6	4.898	16.9	0.040	0.92
	86	2042.1	4.897	17.4	0.042	0.90
	95	2042.4	4.896	17.4	0.042	0.82
OCS:CO:CH_3OH 1:30:10	15	2041.6	4.898	21.0	0.050	1.00
	21	2040.4	4.901	21.0	0.050	1.00
	32	2039	4.904	19.3	0.046	1.00
	44	2040.7	4.900	18.3	0.044	0.93
	56	2041.2	4.899	17.4	0.042	0.92
	66	2041.4	4.899	17.1	0.041	0.92
	75	2041.6	4.898	17.1	0.041	0.91
	86	2042.1	4.897	17.4	0.041	0.90
	95	2042.4	4.896	17.6	0.042	0.83
OCS:CO:CH_3OH 1:40:10	15	2044.7	4.891	20.5	0.049	1.00
	22	2040.3	4.901	20.2	0.049	1.01
	31	2038.6	4.905	18.1	0.043	1.01
	40	2040.3	4.901	16.9	0.041	0.92
	51	2040.6	4.901	16.9	0.041	0.93
	61	2040.6	4.901	16.6	0.040	0.93
	72	2040.8	4.900	16.6	0.040	0.93
	84	2041.3	4.899	17.1	0.041	0.92
	95	2041.8	4.898	17.1	0.041	0.83

* indicates that the OCS ν_3 mode in the marked ice has a strongly asymmetric or split (i.e., non-Gaussian) peak profile.

Table S4: Peak positions, FWHMs, and relative integrated absorbances (with respect to the integrated absorbance of each mixture at 15 K) of the OCS $\nu3$ feature in tertiary H_2S-containing mixtures at various temperatures.

Matrix	T (K)	Peak cm^{-1}	Peak μm	FWHM cm^{-1}	FWHM μm	Rel. integ. abs. (w.r.t. 15 K)
$OCS:CO:H_2S$ 1:5:1*	15	2045	4.890	20.0	0.048	1.00
	27	2044.5	4.891	21.2	0.051	0.98
	33	2042.8	4.895	27.2	0.065	0.92
	38	2037.1	4.909	34.0	0.082	0.89
	48	2034.2	4.916	36.4	0.087	0.90
	57	2030.8	4.924	44.4	0.107	0.85
$OCS:CO:H_2S$ 1:20:1*	15	2050	4.880	18.3	0.044	1.00
	24	2040	4.900	18.6	0.044	1.01
	38	2040	4.900	16.4	0.039	0.93
	44	2040	4.900	47.7	0.114	0.80
	51	2040	4.890	50.9	0.121	0.78
	57	2040	4.900	57.6	0.137	0.79
$OCS:CO:H_2S$ 1:20:5*	15	2046.9	4.885	17.4	0.041	1.00
	29	2042.8	4.895	19.3	0.046	1.00
	37	2040.9	4.900	19.0	0.046	0.98
	42	2040	4.902	29.7	0.071	0.82
	51	2040	4.902	39.1	0.093	0.81
	59	2040	4.902	52.6	0.125	0.82
$OCS:CH_3OH:H_2S$ 1:20:10	15	2041.4	4.899	17.4	0.042	1.00
	50	2041.2	4.899	16.2	0.039	0.99
	60	2041.4	4.899	15.4	0.037	0.99
	80	2041.6	4.898	14.5	0.035	0.98
	96	2041.9	4.897	13.5	0.032	0.86

* indicates that the OCS ν_3 mode in the marked ice has a strongly asymmetric or split (i.e., non-Gaussian) peak profile.

Table S5: Peak positions, FWHMs, and relative integrated absorbances (with respect to the integrated absorbance of each mixture at 15 K) of the OCS $\nu3$ feature in tertiary H_2S-containing mixtures at various temperatures.

Matrix	T (K)	Peak cm^{-1}	Peak μm	FWHM cm^{-1}	FWHM μm	Rel. integ. abs. (w.r.t. 15 K)
OCS:CH_3OH:H_2O 1:20:5	15	2042	4.897	19.5	0.047	1.00
	50	2042.2	4.897	18.1	0.043	0.99
	60	2042.5	4.896	17.4	0.042	0.98
	72	2042.7	4.895	16.9	0.040	0.97
	86	2043	4.895	15.9	0.038	0.96
	101	2043.2	4.894	14.9	0.036	0.85
OCS:CH_3OH:H_2O 1:20:10	15	2042.5	4.896	18.8	0.045	1.00
	50	2042.7	4.895	17.4	0.042	0.99
	65	2043	4.895	16.4	0.039	0.98
	75	2043.2	4.894	15.9	0.038	0.97
	87	2043.5	4.894	15.2	0.036	0.95
	101	2043.5	4.894	14.2	0.034	0.87
OCS:CH_3OH:H_2O 1:20:20	15	2043.2	4.894	19.0	0.046	1.00
	50	2043.2	4.894	17.6	0.042	0.99
	60	2043.5	4.894	17.1	0.041	0.98
	71	2043.7	4.893	16.4	0.039	0.97
	81	2043.9	4.893	15.7	0.038	0.96
	100	2044.2	4.892	14.5	0.035	0.89
OCS:H_2O 1:20	15	2046.6	4.886	23.4	0.056	1.00
	50	2046.8	4.886	21.0	0.050	0.98
	66	2047.1	4.885	19.0	0.045	0.97
	89	2047.3	4.884	17.1	0.041	0.95
	101	2047.3	4.884	15.9	0.038	0.88

* indicates that the OCS ν_3 mode in the marked ice has a strongly asymmetric or split (i.e., non-Gaussian) peak profile.
